# Immune-checkpoint inhibition for tumor prevention in a preclinical Lynch syndrome model

**DOI:** 10.1016/j.tranon.2025.102472

**Published:** 2025-07-16

**Authors:** Annabell Wolff, Johanna Maennicke, Maja Huehns, Paula Krone, Sonja Oehmcke-Hecht, Caterina Redwanz, Wendy Bergmann-Ewert, Christian Junghanss, Claudia Maletzki

**Affiliations:** a Department of Medicine, Clinic III –Hematology, Oncology, Palliative Medicine, Rostock University Medical Center, University of Rostock, 18057 Rostock, Germany; b Institute of Pathology, Rostock University Medical Center, University of Rostock, 18057 Rostock, Germany; c Institute of Medical Microbiology, Virology and Hygiene, Rostock University Medical Center, University of Rostock, 18057 Rostock, Germany; d Department of Internal Medicine B, Cardiology, University Medicine Greifswald, Germany; e Core Facility for Cell Sorting & Cell Analysis, Rostock University Medical Centre, 18057, Rostock, Germany

**Keywords:** T cell exhaustion, Tumor preventive strategies, Immune modulation, Germline MMR mutation carriers

## Abstract

**Background:**

FDA-approved immune checkpoint inhibitors (ICIs) are the state-of-the-art treatment for mismatch-repair deficient tumors (dMMR). Their immunomodulatory effects in a preventive setting, are poorly studied. In this study, we used two ICIs against PD-L1 or LAG-3 in a preclinical mouse model of dMMR-driven carcinogenesis.

**Methods:**

Msh2^loxP/loxP; TgTg(Vil1-cre^ mice without clinical signs of tumor development (i.e. age <12 weeks) received repeated applications (*n* = 8, 28-day interval) of anti-PD-L1, anti-LAG-3 (2.5 mg/kg bw, i.p.) or isotype (anti-IgG1, 2.5 mg/kg bw, i.p.). Blood phenotyping, tumor microenvironment, and hematopoiesis, and presence of procoagulant extracellular vesicles (EV) were studied.

**Results:**

Prophylactic ICI application significantly prolonged overall survival (OS) of Msh2^loxP/loxP; TgTg(Vil1-cre^ mice (OS: anti-PD-L1: 54.4 wks and anti-LAG-3: 58.4 wks vs. ctrl 35.8 wks). Circulating exhausted and regulatory T cells were significantly lower in the ICI groups. Splenic exhaustion markers showed correlating results. Outgrowing tumors showed an inflammatory response-related gene signature. Accompanying immunofluorescence confirmed data and identified reduced numbers of tumor-infiltrating regulatory granulocytes in late-onset tumors. Alterations in bone marrow hematopoiesis accompanied this massive immune modulation, indicating successful prevention of myeloid-shifted hematopoiesis. Plasma coagulation of EVs was not significantly altered in the ICI groups.

**Conclusion:**

Preventive ICI prolongs overall survival of cancer-prone mice. The sustained immune modulation and normal bone marrow hematopoiesis may pre-sensitize late-onset dMMR tumors to a second round of immunotherapy. Hence, we highlight the importance of preventive strategies for germline MMR mutation carriers and recommend improved screening of patients eligible for prophylactic ICIs to improve long-term outcomes.

## Highlights

• Patients with germline mismatch repair mutation have a high tumor risk during their lifetime.
• Preclinical studies proposed the benefit of prophylactic approaches to delay tumor.
• Prophylactic immune checkpoint blockade prolongs survival in a preclinical model.
• LAG-3 blockade is superior to PD-1 blockade, triggering an inflammatory signature.
• LAG-3 blockade prevents myeloid-shifted bone marrow hematopoiesis.

## Introduction

Mismatch repair deficiency (dMMR) is paradigmatic for tumor immunogenicity. This is due to the exceptionally high number of somatic mutations encoding immunogenic neoantigens [[1], [2], [3]], which are foreign to the immune system and can be recognized by cytotoxic T cells [[4], [5], [6]]. Tumors arising in the context of dMMR are therefore prone to be treated with immunotherapy. They develop sporadically or as part of an inherited disease called Lynch syndrome (LS). In the latter, patients are predisposed to early-onset gastrointestinal and extraintestinal tumors.

Germline mutations in MMR genes *Mlh1* or *Msh2* account for approximately 70 % of all cases. Molecularly, a second somatic hit in the remaining allele of the affected gene drives dMMR and initiates the vicious cycle of malignancy. During tumor growth, single mutated cell clones acquire immune evasion and the ability for uncontrolled growth. The immune system's reactivation is a thriving strategy to combat dMMR-driven malignancies. Accordingly, several clinical trials have shown an outstanding response to immune checkpoint inhibition (ICI) in dMMR patients [[7], [8], [9]]. This has led to the first tissue-agnostic treatment approval by the U.S. FDA based on the molecular marker dMMR [10,11]. Hence, ICI treatment has revolutionized dMMR patient management and remarkably improved outcomes. ICIs are often given alone or together with cytostatic drugs in adjuvant and neoadjuvant settings [[12], [13], [14], [15], [16]].

In a huge study of ∼47,000 patients with solid tumors, immunotherapy with ICIs alone and in combination with chemotherapy reduced the incidence of secondary malignancies across different ages and tumor types [17]. Germline mutation analysis was not provided, but this large study was groundbreaking in demonstrating the potential of ICIs not only to exert direct anti-tumor effects but also to induce long-term immunity. However, a recent study did not confirm these promising results in patients with LS harboring pathogenic germline MMR mutations [18]. Despite the excellent overall response rate of dMMR tumors to ICI-based immunotherapy, the prevention of metachronous tumors and pre-neoplastic lesions after ICI in germline mutation carriers has not been demonstrated. However, clinical trials are ongoing to evaluate the potential of ICIs to prevent adenomatous polyps and second primary tumors in patients with LS (*ClinicalTrials.gov ID:* *NCT04711434*, supplementary Table 1).

Another study even reported shared mutated neoantigens as vaccine candidates for LS carriers and affected patients [19]. More recently, an elegant work of Bowen et al. revealed enhanced immune responses through epigenetic reprogramming by EZH2 in the genome of LS mice as a cancer prevention strategy [20], encouraging further emphasis on immune-intercepting strategies.

In this study, we took advantage of the extensive preclinical and clinical evidence given in the therapeutic situation upon ICI application [13,15,[21], [22], [23]]. We examined the potential of prophylactic ICI application using two clinically approved ICIs (anti-PD-L1 and anti-LAG-3). Both ICIs were applied as monotherapy to tumor-free Msh2^loxP/loxP; TgTg(Vil1-cre^ mice and overall survival was analyzed along with a longitudinal assessment of immunologic changes in the periphery and bone marrow.

## Material & methods

### Ethical statement

The German local authority approved all animal experiments: Landesamt für Landwirtschaft, Lebensmittelsicherheit und Fischerei Mecklenburg‐Vorpommern (7221.3‐1‐062/19), under the German animal protection law and the EU Guideline 2010/63/EU. Mice were bred in the animal facility of the University Medical Center in Rostock under specific pathogen‐free conditions. Msh2^loxP/loxP; TgTg(Vil1-cre)^ (briefly: Msh2^loxP/loxP^) genotyping was done as described before [24]. During their whole lifetime, all animals received enrichment in the form of mouse-igloos (ANT Tierhaltungsbedarf, Buxtehude, Germany), nesting material (shredded tissue paper, Verbandmittel GmbH, Frankenberg, Deutschland), paper roles (75 × 38 mm, H 0528–151, ssniff‐Spezialdiäten GmbH), and wooden sticks (40 × 16 × 10 mm, Abedd, Vienna, Austria). During the experiment, mice were kept in type III cages (Zoonlab GmbH, Castrop‐Rauxel, Germany) at 12‐h dark: light cycle, the temperature of 21 ± 2 °C, and relative humidity of 60 ± 20 % with food (pellets, 10 mm, ssniff‐Spezialdiäten GmbH, Soest, Germany) and tap water ad libitum. When mice were subjected to treatment (= age <12 weeks), they were given daily-prepared soaked pellets to ensure proper food intake.

### Experimental protocol

Msh2^loxP/loxP^ mice without clinical signs of tumor development and aged <12 weeks received repetitive prophylactic applications of the ICIs, i.e. anti-PD-L1 or anti-LAG-3 (2.5 mg/kg bw, i.p., *n* = 8 injections in total, 28-day interval, *n* = 11 mice/group). Control mice received an isotype antibody (*n* = 16 mice). The general health status was monitored daily. Mice were sacrificed based on human endpoints (weight loss >15 %, pain, changes in social behavior) or when the maximum follow-up time was reached (12 months after the last intervention). Blood, spleen, tumors, and bone marrow were collected for subsequent analyses.

### Immune phenotyping of blood, spleen, and tumor

Blood samples were routinely collected from anesthetized mice (retrobulbar venous plexus) and centrifuged. Plasma was frozen at −80 °C. Spleen and tumor tissues were disaggregated with a 100 µm cell strainer. Single cells were stained with a panel of conjugated monoclonal antibodies (mAb, 0.125 μg to 1.5 μg each) as described before [21]. Flow cytometry measurements were performed on a spectral flow cytometer (3L-Cytek™ Aurora). Data were analyzed using SpectroFlow™ Version 3.3 and FlowJo™ Version 10.6.1.

### Characterization of hematopoietic stem cells

Bone marrow was obtained by flushing the bones. Then, antibody staining was done to characterize hematopoietic stem cells as described [20]. Flow cytometry measurements were performed on a spectral flow cytometer (3L-CytekTM Aurora). SpectroFlowTM version 2.2.0.3 was used for analysis, and FlowJoTM version 10.6.1 was used for data evaluation.

### Nanostring gene expression analysis

Ten 20 µm cryostat sections were collected and RNA was isolated using the RNeasy Mini Kit (Qiagen). The RNA concentration was measured by Nanodrop and was adjusted to a specific concentration (300 ng/reaction). The gene expression analysis was performed by Nanostring analysis as described before using the nCounter PanCancer IO 360™ Panel. Briefly, this panel enables digital profiling of 770 genes that shape the tumor-immune interface and allows for the characterization of pathways relevant to immune response and escape. Quality control, normalization, and data analysis were done by applying the nSolver™ Analysis Software 4.0 including nCounter Advanced Analysis (version 2.0.115). Data are presented as heatmap. The up and down regulation after treatment and in relation to the isotype of various genes was visualized as a volcano plot showing the –log10 p-(value) and the log2 fold change.

### In vitro co-culture approach

The direct effects of anti-LAG-3 and anti-PD-L1 treatments on tumor cells were assessed using a semi-autologous in vitro co-culture system, as previously described (doi: 10.1080/2162402X.2022.2094583). Flow cytometry was used for data collection, quantifying the number of residual cells within a standardized number of fluorescent microsphere beads (1.4 × 10⁵ beads/ml, 10 µm in diameter; Polyscience, Hirschberg an der Bergstrasse, Germany). Measurements were performed on a BD FACSVerse Cytometer (BD Pharmingen), and data analysis was conducted using BD FACSuite software (BD Pharmingen).

### EV quantification, clotting time assay, and tissue factor (TF) elisa

EVs were isolated from 200 µl plasma of mice of all groups. Plasma was thawed, diluted with 1x PBS up to 1 ml, and centrifuged three times at 14,000 x *g* for 30 min. Between every centrifugation step, 900 μl of the supernatant was removed and 1x PBS was added. EVs were resuspended in 100 μl and used for subsequent experiments. Nanoparticle Tracking Analysis (NTA) with NanoSight® Ltd. (Amesbury, GB) was used to determine EV quantity, size, and concentration. The EVs were diluted with 1x PBS and placed in the assembled measurement chamber. In addition, a thermometer was connected to the measurement chamber. During the measurement, five videos of 60 s each were recorded. The mean value of the concentration and the size of the most abundant EV population were calculated from all videos. To exclude particles of 1x PBS, this was also measured and then subtracted from the samples. Additionally, a clotting time assay was performed on a coagulometer at 37 °C. After adding 100 µl plasma and calcium chloride (CaCl2), the EVs' clotting time was measured. Finally, a mouse TF ELISA (Assay Genie, Biomol, Hamburg, Germany) was applied according to the manufacturer’s instructions to detect TF in the plasma.

### Sorting of MDSCs from total splenocytes and reactive oxygen species (ROS)-Production

ROS Production of total splenocytes and sorted MDSCs (CD11b^+^GR-1^+^ cells) from isotype, anti-LAG3, and anti-PD-L1 exposed Msh2^loxP/loxP^ mice were analyzed as described before [21].

### Immunofluorescence & HE histology

The tumor microenvironment was examined as described before [21,25]. Additionally, liver resection specimens were stained using conventional H&E histology.

### Statistics

GraphPad PRISM software, version 10.4.1 (GraphPad Software, San Diego, CA, USA) was used for statistical evaluation. The value of significance was set to *p* < 0.05. Data was first tested for normality by conducting the Shapiro-Wilk test. In the case of normality, one-way ANOVA (Tukey´s multiple comparisons) or unpaired *t*-test was accomplished. If normality failed, the Kruskal-Wallis or *U test* was applied. Kaplan Meier survival curves were analyzed using the log-rank (Mantel-Cox) test. In the case of blood phenotyping, outliers were eliminated, when they were above or below the average plus/minus two times the standard deviation.

## Results

### Prophylactic ICI application prolongs overall survival of treatment-naïve msh2^loxp/loxp^ mice

**Fig. 1:**
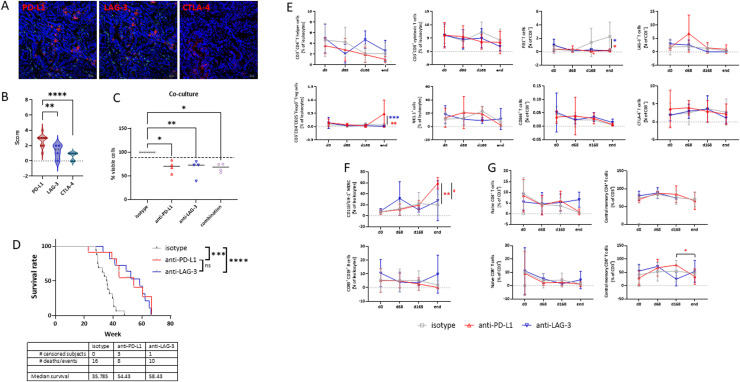
*Ex vivo* analysis on immune checkpoints in primary tumors, overall survival and longitudinal spectral flow cytometry of the peripheral blood from Msh2^loxP/loxP^ mice in a prophylactic immunotherapy setting. (A, B) Abundance of immune checkpoint molecules in murine Msh2^loxP/loxP^-driven tumors. (A) Representative images and (B) quantification by applying a predefined score (0–4). Violin plots + all points are shown; *n* = 4–12 cases/marker, ** *p* < 0.01; **** *p* < 0.0001; one-way ANOVA (Tukey's multiple comparisons test). (C) Semi-autologous in vitro co-culture of murine tumor cells and peripheral blood immune cells. Read out was done by flow cytometry and the % number of residual viable cells is shown. * *p* < 0.05, ** *p* < 0.01 vs. isotype;one-way ANOVA (Tukey's multiple comparisons test). (D) Kaplan Meier survival curve. Msh2^loxP/loxP^ mice without clinical signs of tumor development and aged <12 weeks received repetitive prophylactic applications of the ICIs, i.e. anti-PD-L1 or anti-LAG-3 (2.5 mg/kg bw, i.p., *n* = 8 injections in total, 28 day interval, *n* = 11 mice/group). Control mice received an isotype antibody (*n* = 16 mice/strain). Mice were sacrificed based on human or when the maximum follow-up time was reached (12 months after the last intervention). Log-rank analysis; *p* < 0.0001 vs. isotype; n.s. anti-PD-L1 vs. anti-LAG-3. (E-G) Peripheral blood was taken routinely from retrobulbar venous plexus. A panel of surface and intracellular staining monoclonal antibodies was used and staining was done as described in material and methods. Flow cytometry measurements were performed on a spectral flow cytometer (3L-Cytek™ Aurora). Data were analyzed using SpectroFlo™ Version 2.2.0.3. and FlowJo™ Version 10.6.1. Mean + SD, *n* = 4 – 9 mice/group; * *p* < 0.05; ** *p* < 0.01; *** *p* < 0.001 vs. isotype; ^#^*p* < 0.05 vs. ICI; two-way ANOVA (Tukey's multiple comparisons test).

First, we screened treatment-naïve tumors for the abundance of target proteins. Quantitative analysis of PD-L1, LAG-3, and CTLA-4 expression revealed distinct patterns across the tumors, with a relatively uniform abundance of PD-L1 and LAG-3, but absence of CTLA-4 on tumor-infiltrating T cells (Fig. 1A, B). Additional in vitro co-culture experiments with tumor cells and peripheral immune cells, either with or without immune-checkpoint blockade with anti-PD-L1, anti-LAG-3, or a combination of both confirmed the therapeutic activity of these antibodies (Fig. 1C). Notably, the number of residual tumor cells significantly decreased following treatment with either monotherapy, with similar effects observed between the two single-agent treatments. However, dual blockade of PD-L1 and LAG-3 did not result in a significant enhancement of the therapeutic response, and the number of residual tumor cells remained comparable to that observed with single-agent treatment. Based on this finding and our previous results with therapeutic ICI treatment in vivo, including anti-LAG3 antibody in dMMR-driven tumors [21], we investigated the effect of prophylactic ICI application in the Msh2^loxP/loxP; TgTg(Vil1-cre)^ mouse model. For this purpose, mice under 12 weeks of age and without clinical signs of tumorigenesis received repetitive applications of either the anti-PD-L1 or anti-LAG-3 antibody. Both ICIs significantly prolonged overall survival (OS, Fig. 1D). Compared to the isotype control, OS was extended by 15 weeks in mice receiving the anti-PD-L1 antibody and by 17 weeks in those receiving the anti-LAG-3 antibody (isotype: 35.8 wks vs. anti-PD-L1: 50.0 wks; isotype: 35.8 wks vs. anti-LAG-3 antibody 52.6 wks).

### Prophylactic ICI application has a minor impact on peripheral immune status

Blood was collected longitudinally and analyzed using an in-house immune phenotyping panel to detect potential changes in peripheral immune cell subsets following ICI treatment (Fig. 1E-G). Focusing on the T cell subset, we observed that both CD3^+^CD4^+^ *T* helper cells and CD3^+^CD8^+^ cytotoxic T cells were slightly reduced in anti-PD-L1-treated mice compared to the isotype control. In contrast, T helper and cytotoxic T cell levels showed modest increases in anti-LAG-3-treated mice. At the experimental endpoint, regulatory T cells were significantly reduced in both treatment arms (anti-PD-L1 vs. isotype, *p* < 0.01; anti-LAG-3 vs. isotype, *p* < 0.001, Fig. 1E). The number of NK1.1^+^ cells was unaffected by either ICI.

Assessment of T cell exhaustion markers such as PD1, CD366, LAG-3, and CTLA-4 revealed significant changes only in the fraction of CD3^+^PD1^+^ cells, which were significantly lower in both groups compared to the isotype control (*p* < 0.05). Besides, we identified changes in circulating myeloid cell numbers under ICI treatment (Fig. 1F). CD11b^+^GR-1^+^myeloid-derived suppressor cells (MDSCs) increased in anti-PD-L1 treated mice compared to the isotype control (*p* < 0.01). However, in mice receiving anti-LAG3, no such increase in MDSCs was seen and levels remained significantly lower than in anti-PD-L1 treated mice (*p* < 0.05). While CD83^+^CD19^+^ *B* cell numbers fluctuated slightly, no significant differences were observed between ICI-treated and isotype control groups (Fig. 1F). We next assessed T cell differentiation and quantified the numbers of naïve and central memory CD4^+^ and CD8^+^ *T* cells (Fig. 1G). In mice receiving prophylactic LAG-3 blockade, the number of naïve and central memory CD4^+^ *T* cells remained stable until the experimental endpoint. In contrast, both populations slightly decreased in the other groups (i.e. anti-PD-L1, isotype). Naïve CD8^+^ *T* cells declined across all groups throughout the treatment period, reaching their lowest levels at the endpoint. Central memory CD8^+^ *T* cells showed fluctuating patterns in the anti-LAG-3 group, with an initial increase in mice treated with the anti-PD-L1 antibody. However, by the time of tumor development (i.e., the endpoint), central memory CD8^+^ *T* cell numbers had dropped and were even lower than in control mice.

### Prophylactic anti-LAG-3 application is superior to anti-PD-L1 immunotherapy in modulating the immune phenotype in the spleen

**Fig. 2:**
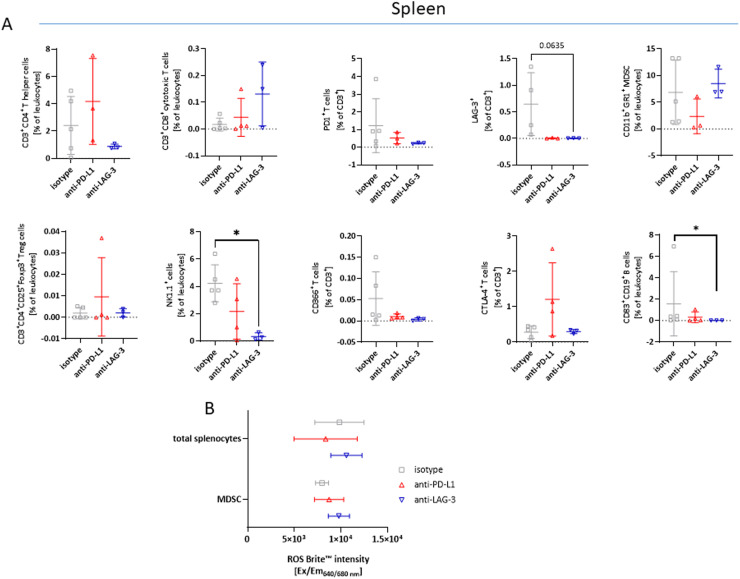
Spectral flow cytometry of spleens. (A) Given is the number of % immune cells at the experimental endpoint resulting from 100,000 events measured on a spectral flow cytometer (3L-Cytek™ Aurora). Mean + SD. (A) *n* = 4 – 9 mice/group; * *p* < 0.05; ** *p* < 0.01 vs. control; Kruskal Wallis test. (B) CD11b^+^GR-1^+^sorted splenocytes were stimulated over night with lipopolysaccharide from *E. coli* (100 ng/ml). ROS production from unsorted and CD11b^+^GR-1^+^sorted splenic MDSCs was determined using ROS Brite™ 670. Fluorescence intensity was measured on a preheated Tecan plate reader. Mean + SD. One-way ANOVA (Tukey's multiple comparisons test).

Tumor-induced immunological changes in the spleen that may reflect systemic immune alterations caused by tumor progression, were analyzed by the same in-house immune phenotyping panel (Fig. 2A).

The quantity and distribution of T helper cells and cytotoxic T cells were not significantly altered by ICI treatment. Similarly, regulatory T cell numbers remained comparable to the isotype control. Cells expressing T cell exhaustion markers such as PD-1 and LAG-3 decreased in both treatments. However, CTLA4^+^ *T* cells increased with anti-PD-L1 treatment, though with high variation between individual samples. Both ICIs led to a reduction of NK1.1^+^ cells and CD83^+^CD19^+^ *B* cells, with the decrease reaching statistical significance in the anti-LAG3 group (*p* > 0.01). The number of splenic MDSCs was slightly decreased by anti-PD-L1, but not by anti-LAG3 treatment. To determine whether these quantitative differences also translated into functional effects, we examined the release of reactive oxygen species (ROS), immunosuppressive cellular byproducts, that contribute to tumor progression. ROS levels were quantified from total splenocytes and CD11b^+^GR-1^+^ sorted MDSCs to identify the primary source of ROS. Overall, ROS production was comparable between treatment groups (Fig. 2B). In MDSCs, both treatments led to a slight increase in ROS production, implying that functional counter-regulation may compensate for differences in MDSC numbers.

### Prophylactic ICI treatment mediates long-term follow-up immune-modulating effects in dMMR tumors

**Fig. 3:**
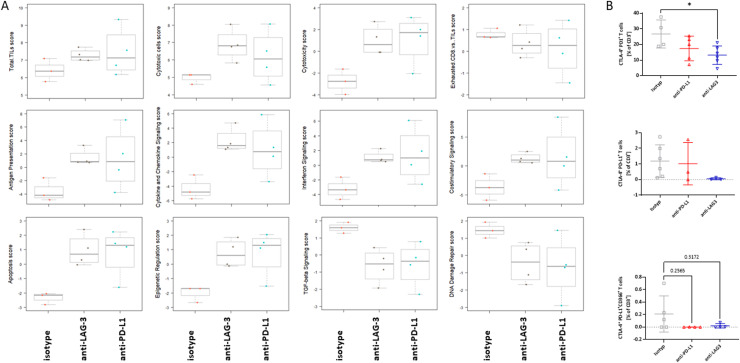
Nanostring gene expression analysis. The nCounter PanCancer IO 360™ Panel was applied. Relative abundances measuring various differences between cell types reported for each group. Data result from *n* = 3–4 samples/group. Heatmap showing changes in specific cell types or signaling pathways according to gene expression data in mice receiving either the isotype or ICIs. (B) Extended Spectral flow cytometry of tumor-infiltrating immune cells. Given is the number of % T cells at the experimental endpoint resulting from 100,000 events measured on a spectral flow cytometer (3L-Cytek™ Aurora). Mean + SD. (A) *n* = 3 – 6 mice/group; * *p* < 0.05; vs. isotype; One-way ANOVA (Tukey's multiple comparisons test).

**Fig. 4:**
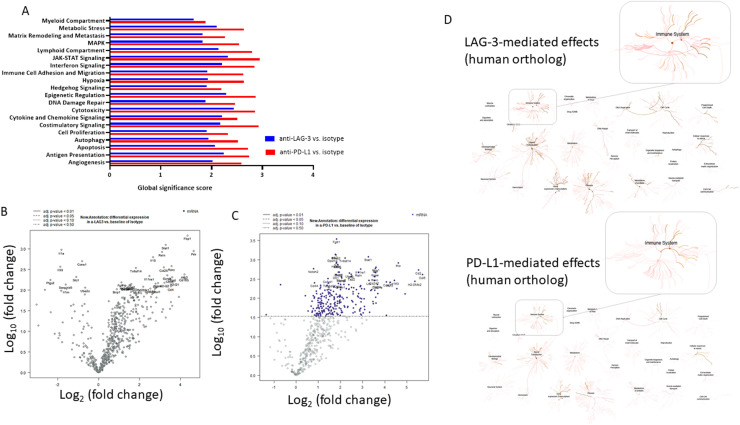
Nanostring gene expression analysis. The nCounter PanCancer IO 360™ Panel was applied. (A) The results of differential expression testing are summarized at the gene set level. Each gene set's most differentially expressed genes are identified, and the extent of differential expression in each gene set is summarized using a 'global significance score’. (B, C) Volcano plots. (B) anti-PD-L1 vs. isotype; (C) anti-LAG-3 vs. isotype. Genes are tested for differential expression in response to each selected covariate. For each gene, a single linear regression is fit using all selected covariates to predict expression. (D) Reactome analysis to visualize the pathways and processes affected by ICI treatment, interpreted in the context of human biology through orthologous relationships.

Subsequent investigation focused on the impact of prophylactic ICI administration on the TME (Fig. 3, 4). Nanostring gene expression analyses demonstrated that both ICIs increased the numbers of tumor-infiltrating lymphocytes (TILs), specifically cytotoxic cells (i.e. *Mx1*, anti-LAG-3 *p* < *0.01;* anti-PD-L1 *p* < 0.0001 vs*.* isotype), while decreasing CD8 exhaustion, thereby inducing long-term immunity. Accompanying flow cytometric analysis of tumor-infiltrating T cells confirmed lower exhaustion levels (Fig. 3B). Notably, numbers of CTLA-4^+^PD1^+^ and CD366^+^PD-L1^+^T cells were significantly lower in LAG-3 treated mice (*p* < 0.05 vs. isotype). Additional long-term effects of preventive ICI treatment included enhanced antigen presentation and costimulatory signaling, as well as upregulation of apoptosis and epigenetic regulation score. In contrast, gene associated with TGF-β signaling and DNA repair were downregulated (Fig. 3A).

The plot shown in Fig. 4 illustrates the differential regulation of various immune-associated signaling pathways compared to the isotype control (Fig. 4A, supplementary Figure 1). Specifically, anti-LAG-3 treatment resulted in sustained activation of the JAK-STAT signaling pathway and the related interferon signaling pathway, as identified by *Stat1* upregulation. Additionally, genes involved in cell migration (*Reln*), metabolic stress (*Fbp1*), Th17 cell differentiation (*Rorc*), and T cell co-stimulation (*Tnfrsf14*) were upregulated in tumors that developed following LAG-3 prophylaxis (Fig. 4B, supplementary Figure 1). Anti-PD-L1 treatment led to an activation of pathways related to antigen presentation, cytokine and chemokine signaling, T cell activation, and angiogenesis (Fig. 4C). Notably, up-regulated genes, included *H2-DMb2* for antigen presentation, *STAT-1* for the JAK-STAT signaling pathway, *Cx3cl*, which regulates the cytokine and chemokine signaling pathway, *Cd2* for T cell activation, and *Fgfr1* for angiogenesis (Fig. 4C). Thus, activation of the JAK/STAT signaling pathway may serve as a general response to checkpoint blockade, contributing to global immune activation. Reactome analysis was subsequently used to gain biological insights into the pathways and processes affected by ICI treatment, interpreted in the context of human biology through orthologous relationships (Fig. 4D). Using the top 20 deregulated genes identified by Nanostring analysis, we confirmed that ICI treatment likely activates the immune system and triggers cellular responses to stimuli, as reflected by the observed gene deregulation in our mouse model.

**Fig. 5:**
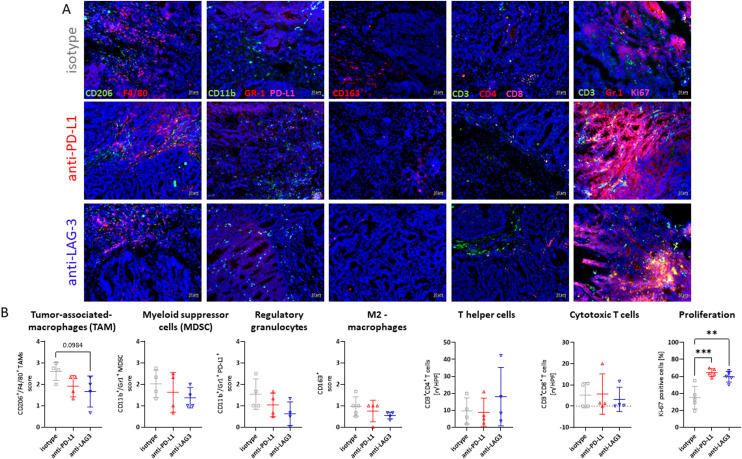
Impact of prophylactic ICI application on the tumor microenvironment. (A) Representative images of dMMR tumor slides for each group and staining. (B) Quantitative analysis of different immune cell types. *n* = 3–4 mice/group; Mean + SD, ** *p* < 0.01; *** *p* < 0.001 vs. isotype; one-way ANOVA (Tukey's multiple comparisons test).

To confirm transcriptional changes on protein levels further, immunofluorescence was performed using whole tumor slides (Fig. 5). Representative images of the entire slides, shown in Supplementary Figure 2, illustrate the distribution of immune cells within the tumors. For the quantification analysis, sections from these whole tumor slides were included, with careful consideration given to intratumoral heterogeneity. However, the impact of such heterogeneity was mostly negligible. With this analysis, we partially confirmed the pre-sensitizing and immune-modulating effect of preventive ICI application. Late-onset dMMR tumors harbored slightly lower numbers of infiltrating Tumor-associated macrophages (TAMs), MDSCs, and regulatory granulocytes. Similarly, infiltration with CD3^+^CD4^+^ helper and CD3^+^CD8^+^ cytotoxic cells was also only marginally higher compared to the isotype control. Furthermore, a significant increase in cell proliferation was observed after ICI prophylaxis, as evidenced higher amounts of Ki67^+^ cells (Fig. 5). Although this may indicate rapid proliferation, as a likely result of higher neoantigen load, it may be still associated with a good response to immunotherapy.

Summarizing the long-term effect of prophylactic ICI immune presensitization revealed preservation of immune activation, although both ICIs exert their effects through different signaling pathways. Anti-LAG-3 outperforms anti-PD-L1 in T cell activation and interferon signaling pathway. Conversely, anti-PD-L1 may enhance immune cell infiltration through pronounced activation of cytokine and chemokine signaling pathways, as well as antigen presentation.

### Prophylactic ICI has long-term effects on bone marrow hematopoiesis

**Fig. 6:**
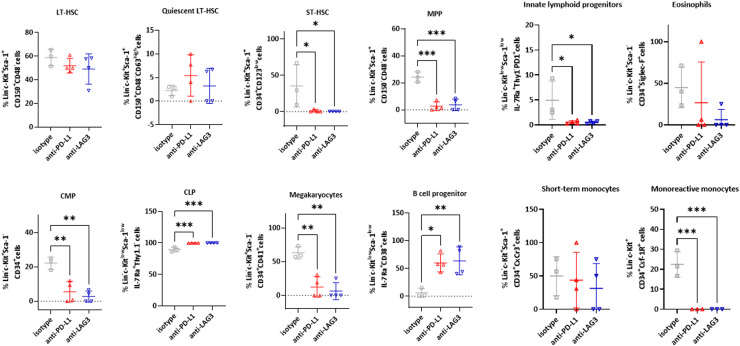
Impact of prophylactic ICI application on murine bone marrow haematopoiesis. A 20-marker panel was applied as described in material & methods. Shown is the quantitative analysis of lin-*c*^−^Kit^+^ precursors according to a predefined characterization scheme [21]. Results show data from 100,000 cells/sample. *n* = 3–4 mice/group; Mean + SD, * *p* < 0.05; ** *p* < 0.01; *** *p* < 0.001 vs. isotype; one-way ANOVA (Tukey's multiple comparisons test).

An imbalanced hematopoiesis is a consequence of tumor growth and proliferation. Here, we studied the effects of prophylactic ICI application using an in-house hematopoietic stem cell full spectrum flow cytometer panel [21] (Fig. 6). While neither ICI affected Lin^−^*c*-Kit^+^Sca-1^+^CD150^+^CD48^−^ long-term hematopoietic stem cells (LT-HSC) and CD63^high^ quiescent LT-HSC, we identified significant changes on numbers of Lin^−^*c*-Kit^+^Sca-1^+^CD34^+^CD123^low^ short-term hematopoietic stem cells (ST-HSC) and Lin^−^*c*-Kit^+^Sca-1^+^CD150^−^CD48^−^ multipotent progenitor cells (MPP). Notably, both populations were barely detectable in mice pretreated with anti-LAG3 (ST-HSC: *p* < 0.05; MPP: *p* < 0.001). Anti-PD-L1 exhibited comparable effects with significantly lower numbers of MPP and a reduction in ST-HSC (Fig. 6).

Additional long-term effects of anti-LAG-3 treatment included a significant decrease in Lin^−^*c*-Kit^+^Sca-1^−^CD34^+^ common myeloid progenitors (CMP), which was counterbalanced by an increase in Lin^−^*c*-Kit^low^Sca-1^low^IL7Ra^+^Thy1^−^ common lymphoid progenitors (CLP). Anti-PD-L1 tended to induce similar effects, implying a shift in the CMP:CLP ratio toward CLP in both treatment groups. This shift correlated with a decrease in Lin^−^*c*-Kit^+^Sca-1^−^FcyRII/III^−^CD34^+^ megakaryocytes and an increase in B-cell progenitors, both of which were significant following anti-LAG3 treatment (Fig. 6). Another notable finding was the sustained reduction of Lin^−^*c*-Kit^low^Sca-1^low^IL7Rα^+^Thy1^−^PD1^+^ innate lymphoid progenitors in both ICI groups. Short-term CxCr3^+^ granulocyte-macrophage progenitor (GMP)-derived monocytes were not affected, but the number of monoreactive Csf-1R^+^ monocytes was significantly decreased in both treatment groups, further supporting a positive shift toward enhanced lymphoid hematopoiesis.

### Prophylactic ICI application does not alter extracellular vesicle (EV) secretion but triggers systemic coagulation

**Fig. 7:**
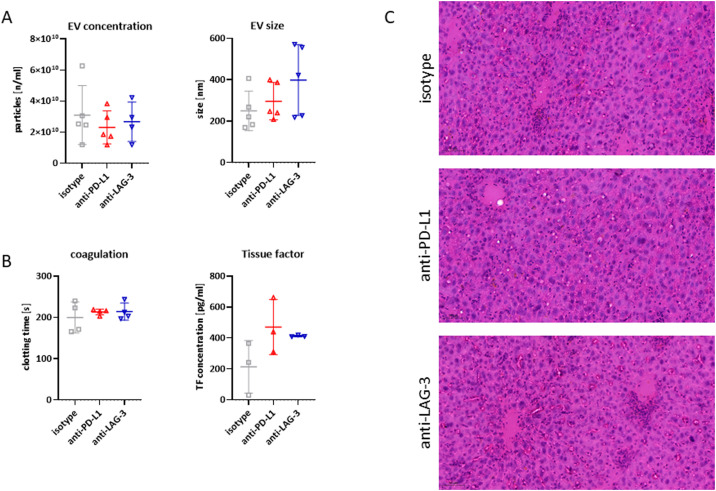
Characterization, coagulation, and TF amount of EVs. (A - B) EVs were isolated from the plasma of Msh2^loxP/loxP^ mice. Their concentration and size were analyzed by NTA and the clotting time was determined. Plasma from both mouse strains was analyzed for TF concentration using a TF ELISA kit. *n* = 3 mice/group; Mean + SD; one-way ANOVA (Tukey's multiple comparisons test). (C) Representative HE stainings of liver tissue taken from mice at the experimental endpoint to exclude toxic systemic side effects.

Finally, we examined the amount of extracellular vesicles in plasma and their potential role in mediating a procoagulant state upon ICI pre-treatment (Fig. 7). Although the concentration of EVs did not differ between treatment groups and isotype controls, EV size tended to be larger, particularly in anti-LAG3-treated mice (Fig. 7A). We then investigated whether ICIs induced a procoagulant state by measuring EV clotting times and EV-bound tissue factor (TF) levels (Fig. 7B). A slight prolongation of clotting time was observed in both treatment groups, however, this finding was inconsistent with the amount of circulating TF. In fact, we detected higher TF concentrations, particularly in the EVs of mice treated with anti-PD-L1. Although TF is the primary initiator of the extrinsic coagulation cascade, its increased presence was not associated with a state of hypercoagulability and may therefore have minimal functional relevance – at least in this preclinical model.

To assess potential toxic side effects of preventive ICI application, we conducted histological analysis of liver slides from mice post-mortem (Fig. 7C). This examination confirmed the absence of systemic toxicity, as no signs of necrosis, steatosis, or increased immune infiltration were observed in the liver tissue. Thus, our findings support that prophylactic ICI intervention is both a safe and effective strategy for tumor prevention.

## Discussion

The extended timeframes required for long-term follow-up in cancer prevention trials significantly limit the availability of robust clinical data, which remain scarce and are only occasionally supported by strong evidence. In 2021, Reyes-Uribe *et al*. conducted a pioneering and elegant co-clinical trial demonstrating that naproxen, a nonsteroidal anti-inflammatory drug, can activate resident immune cells within the colorectal mucosa of patients with Lynch syndrome. Their study revealed that naproxen modulates cytokine-associated pathways and enhances immune cell activity, particularly involving T cells, dendritic cells, and macrophages, suggesting a potential role for NSAIDs in immune-mediated cancer prevention [26]. Hence, prophylactic immune-modulating strategies hold promise for preventing or delaying tumor formation.

By targeting two clinically relevant immune checkpoints, i.e. LAG-3 and PD-L1, we successfully prevented early-onset tumor development in the preclinical Msh2^loxP/loxP^ mouse model, significantly prolonging overall survival following repetitive low-dose anti-PD-L1 or anti-LAG-3 administration. A direct comparison of the two inhibitors revealed a slight superiority of anti-LAG-3 over anti-PD-L1, potentially due to the presence of LAG-3^+^ *T*-cells at early tumor stages, whereas PD-L1 plays a more functionally relevant role at later stages. In either case, the prophylactic use of these ICIs has a positive impact on tumorigenesis [[27], [28], [29]]. Through longitudinal monitoring of circulating immune cells and endpoint immune phenotyping of splenic and tumor-infiltrating leukocytes, we observed surprisingly modest immune-modulating effects. The tumor-preventive effects primarily involved the suppression of regulatory T cells and a low level of T cell exhaustion in the periphery (PD1^+^), as well as in the spleen, identified by reduced numbers of LAG-3^+^ splenocytes in both treatment groups. However, this positive immune-modulating effect was counteracted by a significant increase in circulating MDSCs, particularly after anti-PD-L1 treatment. Similar to regulatory T cells, MDSCs exert their immunosuppressive effect *via* the secretion of IL-10 and TGFβ [30,31]. This, in turn, promotes tumor growth and eventually impairs clinical response to ICI treatment. In melanoma patients receiving anti-PD-1 or CTLA-4 therapy, circulating MDSC numbers initially increased in responders but subsequently declined. Non-responders showed no such fluctuation, and MDSC levels remained high [[32], [33], [34]]. This fits with our previous findings in preclinical dMMR mouse models, where circulating MDSC numbers always increased over time – regardless of the applied immunotherapy, i.e. ICI, vaccine, or combination strategies, and the outcome, i.e. good or poor response to treatment [21,[35], [36], [37]]. Besides, we observed an inverse correlation between MDSC and CD4^+^ *T* helper cells in both prophylactic settings, likely attributable to the global inhibiting effect of T cell function by MDSC. As a result, MDSCs remain a debated biomarker for predicting ICI treatment response. Also, MDSC levels in the peripheral blood do not always correlate with those in the spleen, a secondary immune organ that facilitates communication between circulating and organ-bound immune cells. Here, we did not see significant differences in MDSC numbers in the spleen. However, functional analysis revealed the persistence of their immunosuppressive phenotype, which was here detected by consistently high levels of reactive oxygen species (ROS). Importantly, ROS should not be considered solely from a pathological perspective; their concentration also plays a physiological role in regulating effector T cell proliferation, differentiation, and activation. Accordingly, splenocytes may actively produce ROS and contribute to immune regulation. This finding further underscores the inverse correlation between MDSCs and immune cells. While MDSCs produced high amounts of ROS, additional signaling pathways appear to be influenced by anti-LAG-3 and anti-PD-L1, resembling mechanisms more akin to inhibitory T cell regulation. Therefore, immune phenotyping should always be complemented with functional analysis to accurately assess immune status.

Another important predictor of response and outcome is the local tumor microenvironment. Our results demonstrated significant differences in the immune compartment between ICI-exposed and control mice. Notably, these differences were detectable long after treatment offset, implying a long-term immune-modulating effect of the ICIs. Anti-LAG-3 treatment resulted in the upregulation of the JAK-STAT, interferon, and NF-κB signaling pathways, indicating enhanced T cell activation and differentiation. This immune modulation likely contributes to an improved anti-tumor response by upregulating MHC class I expression, promoting antigen presentation, inducing Th1 polarization, and increasing both cytotoxic activity and memory T cell formation. Similar mechanisms are likely to occur in the human system, as suggested by the predicted activation of immune responses and induction of cellular responses to stimuli, consistent with the gene deregulation observed in our mouse model.

Using whole tumor slides, a slight decrease in tumor-associated CD163^+^(M2) macrophages and regulatory granulocytes was additionally shown. In contrast, numbers of MDSCs and CD8^+^ cytotoxic T cells were similar to control tumors. Infiltration with CD4^+^ *T* helper cells was higher in dMMR tumors following anti-LAG-3 treatment, underlining the superior long-term immune-modulating effect after targeting this immune checkpoint. Aside from this study, it must be acknowledged that the composition of the TME to prophylactic approaches with ICI is a relatively understudied area, particularly in the context of dMMR-related cancer. A recent study from 2023 offers novel insights and reveals that preventive administration of anti-PD-1 therapy led to a significant delay of esophageal carcinogenesis, accompanied by substantial augmentation of memory T cell infiltration within the tumor microenvironment [38]. To the best of our knowledge, the efficacy of LAG-3 blockade has not been evaluated in a prophylactic setting before, and we therefore show for the first time that anti-LAG-3 treatment holds promise in both prophylactic and therapeutic [21] settings.

Finally, the long-term immune-modulating effects of prophylactic ICI treatment included alteration of hematopoiesis, thereby influencing blood-borne immunity. The numbers of short-term HSCs and MPP remained lower than in controls, potentially indicating a depleted stem cell reserve due to the increased utilization of immune cells for the immune response. The heightened interferon and cytokine signaling pathways detected within the tumor may also play a role, as these signaling molecules drive progenitor cell differentiation, suggesting a boost-like transition of progenitor cells into peripheral blood cells. The most significant finding was a shift in hematopoiesis toward lymphoid lineage differentiation, as reflected by changes in the CMP:CLP ratio. This shift was marked by an increase in progenitor cells, particularly B cell progenitors, which may indicate an emerging and amplified immune response. This finding correlates with altered B cell numbers in the blood and spleen, especially following anti-LAG-3 treatment. Conversely, myeloid differentiation was affected, leading to a reduction in CMPs and their downstream precursor cells. Notably, a subset of the monocytic lineage appeared to be particularly affected. The CSF-1R signaling pathway plays a pivotal role in monocyte differentiation and expansion. By releasing its ligand, CSF-1—potentially through tumor-mediated mechanisms—Csf-1R⁺ monocytes can differentiate into MDSCs, which then acquire immunosuppressive properties. The observed post-treatment decrease in Csf-1R⁺ monocytes supports this hypothesis, although relatively high MDSC levels were still detected in the blood of anti-PD-L1-treated mice. This suggests that additional signaling pathways may also contribute to these effects. Hypercoagulation, for instance, is a hallmark of late-stage cancer and is associated with an increased risk of thrombosis. Acquired coagulopathy is also a known complication of certain treatment regimens, including chemotherapy. However, the impact of ICIs on coagulation is less well documented, with prior studies yielding conflicting results. Some reports suggest dysregulation of the coagulation-fibrinolysis balance and an elevated thrombosis risk in patients receiving ICIs [[39], [40], [41]]. This is particularly relevant in prophylactic settings, where the goal is to induce long-term effects while minimizing the risk of serious adverse events. In this study, we found no evidence of ICI-driven hypercoagulation. Although our results showed inconsistencies between total clotting times and TF levels, we interpret these findings with caution, as clotting time was assessed in extracellular vesicles, whereas TF concentration was measured in whole plasma. Whether ICIs influence the coagulation profile in human dMMR-related cancer remains unclear, as the mechanisms underlying immune modulation, escape, and activation in dMMR-driven tumorigenesis are not yet fully understood.

## Conclusions

This study underscores the importance of preventive approaches in hereditary cancer syndromes. Using two clinically-approved immune checkpoint inhibitors, we demonstrate that both antibodies effectively delay tumor formation in a preclinical mouse model. The tumor-preventive effects include preventing T-cell exhaustion and preserving normal hematopoiesis by inhibiting a shift toward excessive myelopoiesis. The late-onset outgrowing tumors exhibit an immune-hot microenvironment, characterized by the upregulation of genes involved in antigen presentation, interferon signaling, costimulation, and apoptosis, providing an ideal basis for subsequent clinical translation of this approach as a cancer prevention strategy.

## CRediT authorship contribution statement

**Annabell Wolff:** Writing – original draft, Methodology, Data curation. **Johanna Maennicke:** Formal analysis, Data curation. **Maja Huehns:** Software, Data curation. **Paula Krone:** Formal analysis, Data curation. **Sonja Oehmcke-Hecht:** Data curation. **Caterina Redwanz:** Formal analysis, Data curation. **Wendy Bergmann-Ewert:** Data curation. **Christian Junghanss:** Writing – review & editing. **Claudia Maletzki:** Writing – review & editing, Validation, Supervision, Formal analysis, Conceptualization.

## Declaration of competing interest

The authors declare that they have no conflict of interests.

## Appendix Supplementary materials

Image, application 1

Image, application 2

Supplementary Figure 1: Nanostring gene expression analysis. The nCounter PanCancer IO 360™ Panel was applied. Relative abundances measuring various differences between cell types reported for each group. Data result from *n* = 3–4 samples/group. Heatmap showing changes in specific signaling pathways according to gene expression data in mice receiving either the isotype or ICIs. Image, application 3
