# Supplementary material for: Immune-checkpoint inhibition for tumor prevention in a preclinical Lynch syndrome model
Source: Transl Oncol. 2025 Jul 16;60:102472. doi: 10.1016/j.tranon.2025.102472 (PMC12284698; doi:10.1016/j.tranon.2025.102472)
Supplement: Supplementary file 1 [file mmc1.docx]

| **NCT Number** | **Study Title** | **Study Status** | **Interventions** |
| --- | --- | --- | --- |
| NCT04711434 | PD-1 Antibody for The Prevention of Adenomatous Polyps and Second Primary Tumors in Lynch Syndrome Patients | RECRUITING | DRUG: PD-1 Antibody |
| NCT04920149 | Mesalamine for Colorectal Cancer Prevention Program in Lynch Syndrome | RECRUITING | DRUG: Mesalamine\|DRUG: Placebo |
| NCT05419011 | Testing a Combination of Vaccines for Cancer Prevention in Lynch Syndrome | RECRUITING | BIOLOGICAL: Adenovirus 5 CEA/MUC1/Brachyury Vaccine Tri-Ad5\|PROCEDURE: Biopsy Procedure\|PROCEDURE: Biospecimen Collection\|PROCEDURE: Colonoscopy\|DRUG: Nogapendekin Alfa\|DRUG: Placebo Administration\|OTHER: Questionnaire Administration |
| NCT02497820 | Finding the Best Dose of Aspirin to Prevent Lynch Syndrome Cancers | NOT_YET_RECRUITING | DRUG: Aspirin |

**Supplementary Table 1: Overview on current clinical trials aiming at tumor prevention in Lynch Syndrome patients.**
