# Supplementary figures and images for: Immune-checkpoint inhibition for tumor prevention in a preclinical Lynch syndrome model
Source: Transl Oncol. 2025 Jul 16;60:102472. doi: 10.1016/j.tranon.2025.102472 (PMC12284698; doi:10.1016/j.tranon.2025.102472)

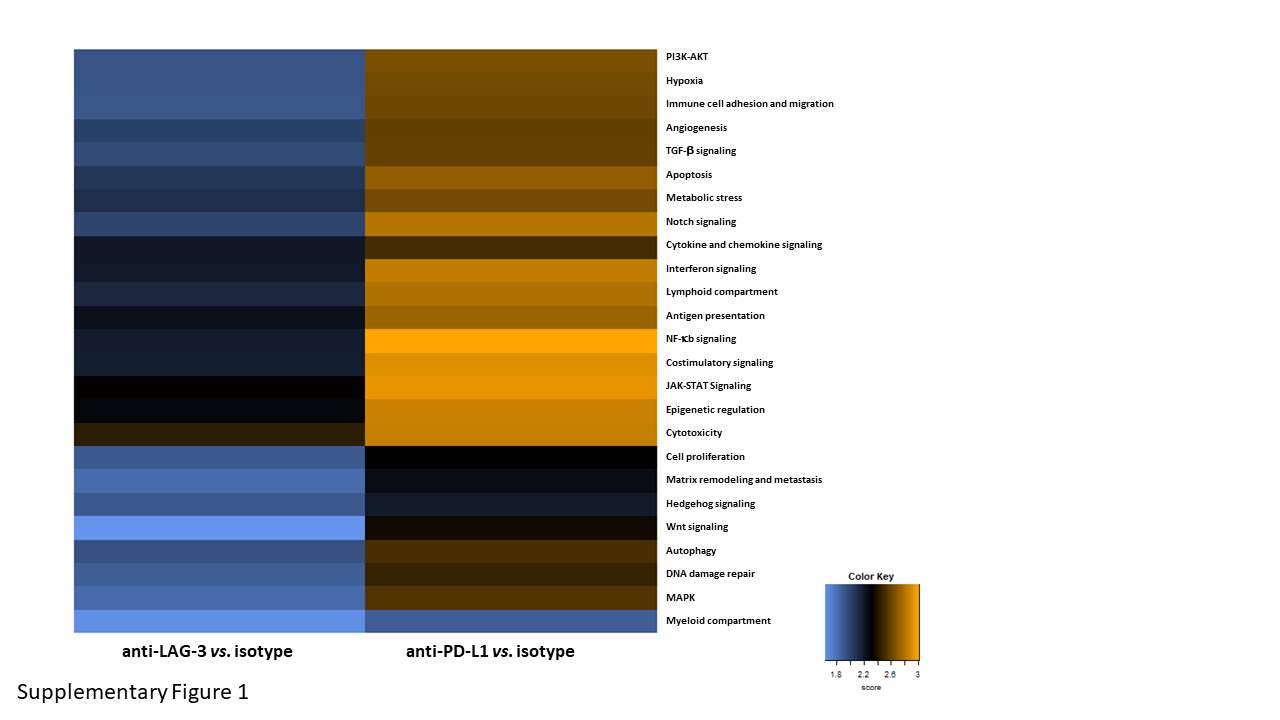

Supplement: Supplementary file 2 [file mmc2.zip › Slide8.TIF]

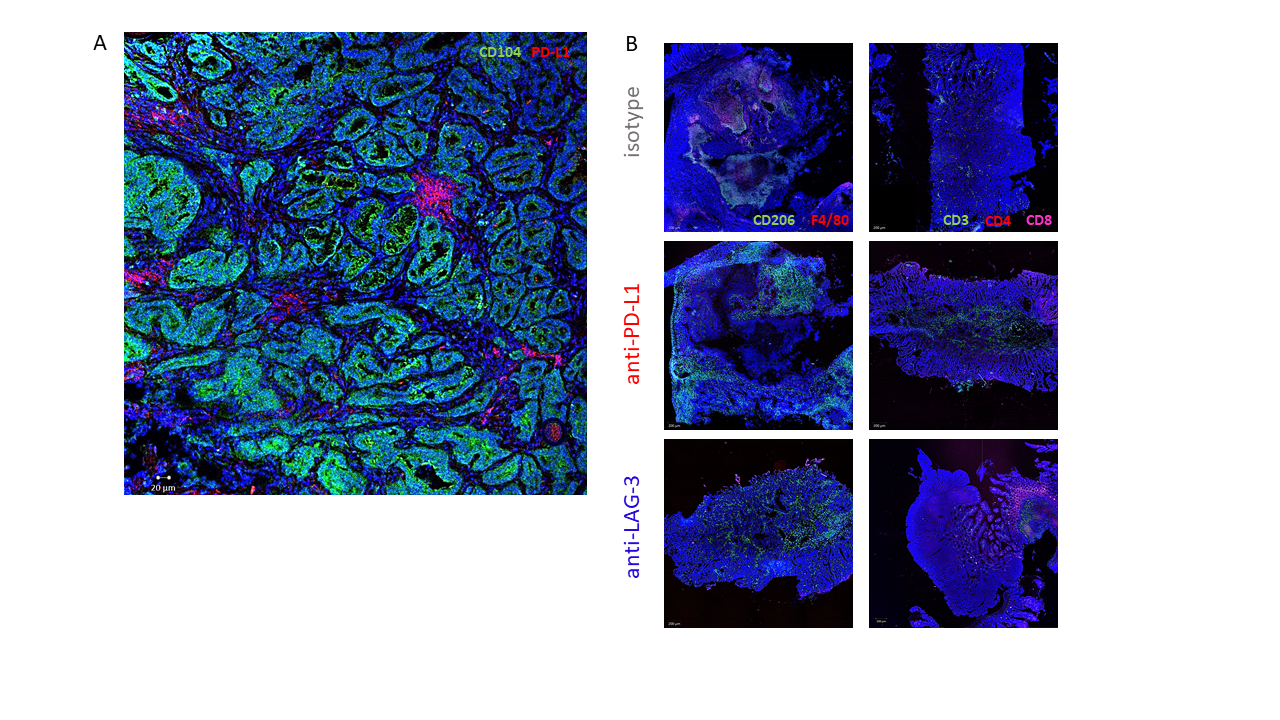

Supplement: Supplementary file 3 — Supplementary Figure 1: Nanostring gene expression analysis. The nCounter PanCancer IO 360™ Panel was applied. Relative abundances measuring various differences between cell types reported for each group. Data result from n = 3–4 samples/group. Heatmap showing changes in specific signaling pathways according to gene expression data in mice receiving either the isotype or ICIs. [file mmc3.zip › Slide9.TIF]
